# Association Analysis of Urotensin II Gene (UTS2) and Flanking Regions with Biochemical Parameters Related to Insulin Resistance

**DOI:** 10.1371/journal.pone.0019327

**Published:** 2011-04-29

**Authors:** María E. Sáez, Tarik Smani, Reposo Ramírez-Lorca, Ignacio Díaz, Manuel Serrano-Ríos, Agustín Ruiz, Antonio Ordoñez

**Affiliations:** 1 Department of Structural Genomics, Neocodex, Sevilla, Spain; 2 Grupo de Fisiopatología Cardiovascular, Instituto de Biomedicina de Sevilla, Hospital Universitario Virgen de Rocío/CSIC/Universidad de Sevilla, Sevilla, Spain; 3 Diabetes Research Laboratory, Biomedical Research Foundation, University Hospital Clínico San Carlos, Madrid, Spain; University of Las Palmas de Gran Canaria, Spain

## Abstract

**Background:**

Urotensin II (UII) is a potent vasoconstrictor peptide, which signals through a G-protein coupled receptor (GPCR) known as GPR14 or urotensin receptor (UTR). UII exerts a broad spectrum of actions in several systems such as vascular cell, heart muscle or pancreas, where it inhibits insulin release.

**Objective:**

Given the reported role of UII in insulin secretion, we have performed a genetic association analysis of the UTS2 gene and flanking regions with biochemical parameters related to insulin resistance (fasting glucose, glucose 2 hours after a glucose overload, fasting insulin and insulin resistance estimated as HOMA).

**Results and Conclusions:**

We have identified several polymorphisms associated with the analysed clinical traits, not only at the UTS2 gene, but also in thePER3 gene, located upstream from UTS2. Our results are compatible with a role for UII in glucose homeostasis and diabetes although we cannot rule out the possibility that PER3 gene may underlie the reported associations.

## Introduction

Urotensin II (UII) is a cyclic undecapeptide that was initially isolated from fish urophysis and subsequently discovered in mammals including humans [1]. UII is considered the most potent endogenous vasoconstrictor discovered to date. It binds to a G-protein coupled receptor (GPCR) known as GPR14 or urotensin receptor (UTR) [2], which is widely expressed in cardiovascular, pulmonary, central nervous, renal and metabolic systems. UII has emerged as a contributor to cardiovascular physiopathology [3], [4].

**Figure 1:**
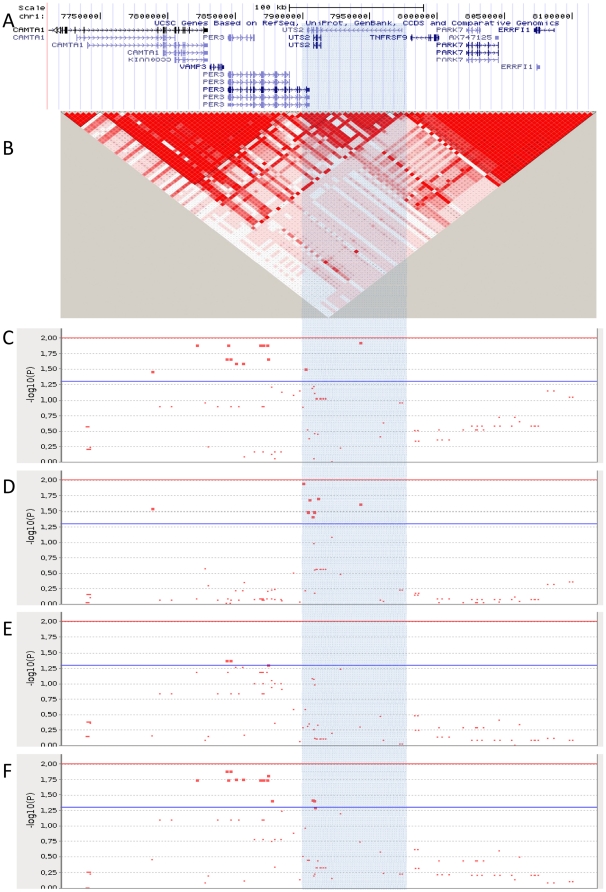
The *UTS2* gene region. A. University of Santa Cruz (UCSC) gene region map. B. Linkage disequilibrium map. C, D, E, F. Graphical representation of the association analysis of the *UTS2* gene region with fasting glucose (C), 2 h-glucose (D), fasting insulin (E) and HOMA (F). Dots symbolize the logarithm of the p value at each marker.

The UTS2 gene, encodes UII and is located at 1p36.23. There are three different UII preproteins encoded by three different transcripts (Figure 1). In accordance with the University of Santa Cruz Genome Browser (http://genome.ucsc.edu/cgi-bin/hgGateway), two of them, named UCSC uc001aos.2 and uc001aor.2, are short mRNA transcripts of 5.8 Kb and 5.5 kb respectively encoding two preproteins of 139 and 124 aminoacids that differ in the amino-terminal end. The third one, called uc001aoq.2, is a longer transcript of about 70 kb that has been identified in pancreas and spleen libraries. It codes for a preproprotein isoform of 139 aminoacids with the same amino-terminal end of the uc001aor.2 transcript and a different carboxi-terminal end. The mature peptide, with only 11 aminoacids, exerts a broad spectrum of actions in several systems through UTR binding. UII promotes cell proliferation of vascular and cancer cells, has inotropic and hypertrophic effects on heart muscle, modulates glomerular filtration and release of catecholamines in the kidney [5]. UII is also present in the pancreas where inhibits insulin release [6], [7]. Elevated plasma levels of UII and increased UII expression have been evidenced in numerous diseased conditions, including hypertension, atherosclerosis, heart failure, pulmonary hypertension, diabetes, renal failure, and the metabolic syndrome [8].

Several polymorphisms at the UTS2 gene have been associated with type 2 diabetes mellitus (T2DM) [9]-[13], insulin resistance [14] and diabetic complications such us diabetic retinopathy and carotid atherosclerosis [15]. The UTS2 gene regulates fat accumulation in skeletal muscle and fatty acid metabolism, two biological pathways related to T2DM in humans [16]. UTS2 polymorphisms have also been associated with essential hypertension [17]. Finally, UTS2 gene polymorphisms are associated with myocardial infarction in individuals with and without metabolic syndrome [18], [19]. All of these associations have been described in Asian populations.

This study is aimed at investigating the implication of UTS2 gene variants in several traits related to T2DM in the Spanish population. For this purpose, we have extracted genotypic data at the UTS2 gene region from a GWAS recently performed in a Spanish population-based sample [20].

## Results

We have analysed for association with fasting glucose, 2 h-glucose, insulin and HOMA, the region of chromosome 1 from 7,711,426 to 8,168,634 bp, including 97 polymorphisms and seven genes: CAMTA1, VAMP3, PER3, UTS2, TNFRSF9, PARK7 andERRFI1.Regarding the UTS2 region, only one of the SNPs analysed, namely rs228652, is located in the chromosomal region shared by the three UTS2 transcripts. The remaining 21 polymorphisms are located at the region coding for the longest mRNA (uc001aoq.2). Figure 1A shows the genomic organisation in this region.

We have detected several associations for all the phenotypes analysed (Tables S1, S2, S3, S4). These associations are confined to the LD block of UTS2 gene at the adjacent one upstream (D′ between blocks 0.75) (Figure 1B). It is interesting to note that none of them is downstream the LD decay observed at the 3′ end of the region encoding the longest transcript (Figure 1C–D).

For fasting glucose, most of significance is confined to the PER3 gene, the adjacent one upstream UTS2. Two SNPs in the UTS2 region are also associated with glucose levels (rs17374781 and rs665244). A similar situation occurs in the test for association with HOMA, where most of associated SNPs lie within PER3 region and only two (rs504560 and rs500508) are in the UTS2 region. Fasting insulin is the phenotype for which less associated SNPs have been found and all of them are located at PER3 gene. For 2 h-glucose, the pattern of association is slightly different, since we cannot observe a cluster of associated SNPs at the UTS2 gene region.

### Haplotypic analysis

In order to clarify if observed associations could be related to UTS2 locus, we performed a haplotype analysis at this candidate region (Table 1). With this purpose, we selected tagging SNPs. Haplotypes with a population frequency (MHF) under 5% were rejected.

**Table 1:** Haplotypic analysis.

HAPLO	FREQ	BETA(A) GLC	BETA(A) OGTT	BETA(A) insul	BETA(A) HOMA
**GENES: PER3, UTS2**					
CCCTTGCGACAGCC	0.158	(-ref-)	(-ref-)	(-ref-)	(-ref-)
CTCGTATGGTGGTC	0.0604	−0.02054	0.02258	−0.02873	−0.02133
CTCTCGTAATAATC	0.101	−0.035	−0.002814	−0.1448	−0.1575
CCCTTGCGATGGTC	0.109	−0.04498	−0.07108	−0.1303	−0.1592
CTCTCGCGATGGTC	0.0628	−0.003319	0.02066	0.007429	−0.002714
		F = 2.34	F = 1.22	F = 2.91	F = 3.32
		df = 4, 704	df = 4, 598	df = 4, 598	df = 4, 598
		p = 0.0542	p = 0.301	p = **0.0212**	p = **0.0105**
SNPs:rs697690,rs227163,rs10462020,rs228682,rs228641,rs1040397,rs504560,rs228703,rs665244,rs2066978,rs2066980,rs228652,rs170631,rs4908486					
**GENE: UTS2**					
GCTTCCCGC	0.149	(-ref-)	(-ref-)	(-ref-)	(-ref-)
ATTTTGAGC	0.065	−0.03465	−0.01244	0.05278	0.03828
GTCTTGCCC	0.116	−0.03363	−0.02672	−0.07208	−0.09015
GCTCTCCGC	0.0744	0.007965	−0.05835	−0.109	−0.1114
GCTTTGCGC	0.235	−0.02672	−0.02313	−0.03691	−0.05894
ACTTTGACA	0.0615	0.04437	0.05885	−0.1732	−0.15
		F = 3.1	F = 0.971	F = 2.02	F = 1.76
		df = 5, 710	df = 5, 602	df = 5, 602	df = 5, 602
		p = **0.00897**	p = 0.435	p = 0.0738	p = 0.119
SNPs: rs228724,rs228721,rs1040396,rs665244,rs2066978,rs4908486,rs17374781,rs579992,rs228652					
**GENE: PER3**					
CGCATTA	0.374	(-ref-)	(-ref-)	(-ref-)	(-ref-)
CGTGCTA	0.136	−0.007949	0.03718	0.108	0.1068
CTTATTT	0.14	−0.02031	0.03539	−0.07667	−0.08529
CTTATTA	0.175	−0.01644	0.05486	0.0318	0.01328
GGCACTA	0.0555	0.04007	0.08027	−0.1653	−0.1447
		F = 1.81	F = 1.38	F = 3.79	F = 3.05
		df = 4, 708	df = 4, 600	df = 4, 600	df = 4, 600
		p = 0.126	p = 0.24	P = **0.0047**	p = **0.0167**
SNPs: rs696306,rs12741937,rs10864316,rs228682,rs228664,rs2640909,rs170631					

We first selected tagging SNPs for the whole region (from CAMTA1 to ERRFI1). We selected 25 SNPs that determine 557 possible haplotypes, only 3 with a MHF>5%. No overall significance was observed (data not shown).

We next restricted the selection of tagging SNPs to the PER3-UTS2 region. We analysed 14 tagging SNPs that form 224 possible haplotypes, 5 of them with MHF>5%. These haplotypes were associated with fasting insulin (F = 2.91, df = 4, p = 0.021) and HOMA (F = 3.32, df = 4, p = 0.011). For fasting glucose, only a trend for association was observed (F = 2.34, df = 4, p = 0.054).

To further narrow the most associated chromosomal region, we analysed independently PER3 and UTS2 regions. For PER3, 7 tagging polymorphisms determine 5 common haplotypes that modulate fasting insulin (F = 3.79, df = 4, p = 0.0047) and HOMA values (F = 3.05, df = 4, p = 0.017). For UTS2, 9 SNPs were selected to conform 6 common haplotypes; these haplotypes are strongly associated with fasting glucose values (F = 3.1, df = 5, p = 0.008) and show a trend for fasting insulin values (F = 2.02, df = 5, p = 0.074). In order to assess the independence of these observations, we analysed both regions in a single regression model in which haplotypes at one of the loci are tested for association with the study phenotypes entering the tagging SNPs of the others loci as covariates in the model. As expected, controlling one locus by the effect of the other locus, all associations are lost (0.176≤p≤0.945) indicating that the observed associations at both loci are not independent and that they are tracking the same disease allele.

## Discussion

In this report, we have analysed the chromosomal region encoding UTS2±200 kb. Genotypic data derived from a 250 k Affymetrix GWAS in our population was combined with Hapmap data to increase the density of SNPs in the region through imputation. To our knowledge, this is the first time that UTS2 is analysed for association with T2DM related traits in a European population. In the monogenic analysis, positive signals are scattered over PER3 and UTS2 gene regions. The joint analysis of both genes by means of tagging SNPs and haplotypic association analyses showed a modest association for insulin and HOMA, although for glucose values we only observed a trend toward association. Analysing both genes independently we found that PER3 region is strongly associated with fasting insulin and UTS2 with fasting glucose. However, the integration of both regions in a single model in which the effect of one of them is controlled for the effect of the other region indicates that the observed associations at both loci are not independent, but they are related to the same causal site.

Previous studies have associated the UTS2 gene variants with T2DM traits. The 143G>A (rs228648) was associated with T2DM in Chinese [9], [13] and the 3836C>T (S89A, rs2890565) with insulin levels and T2DM in Japanese [10], [11]. In the report by Ong et al. in Hong Kong Chinese population [14], these two polymorphisms and the promoter SNP −605G>A (rs228647) were associated with insulin resistance estimated as HOMA. The study of Ong et al. [14] is the first to analyse and associate a promoter polymorphism with T2DM related traits and also the first to construct haplotypes at the UTS2 gene region; they found that carriers of the three-loci haplotype GGT made up of −605G, 143G and 3836T had higher plasma levels of UII, fasting plasma insulin, HOMA-IR and pancreatic β-cell function estimated as HOMA-β%.

In our study, we have a poor coverage of the UTS2 region coding for the two short transcripts. The rs228652 analysed by our group is located only 849 bp from the S89A polymorphism (D′ = 0.77) but it is not associated with any of the traits analysed in our population. By contrast, we have extended our study to UTS2 region encoding the long transcript and flanking regions and have found that the region associated with T2DM related traits exceeds the UTS2 region and include the PER3 gene. PER3 is one of the period clock genes implicated in the circadian clock function. Alterations of the internal clock function is related to the development of obesity and other metabolic age-related diseases, including abnormal glucose metabolism [21]. PER3 mRNA levels have been shown to be lower in T2DM subjects and to negatively correlate with glycosylated haemoglobin and fasting glucose levels [22], [23]. In addition, a polymorphic 54-bp repeat length variant was associated with higher serum levels of IGF-I and IGF-I to IGFBP3 ratios [24]. This polymorphism has also been associated with IL-6 serum levels [25]. IL-6 is an adipokine, a class of cytokine which includes molecules such as TNFα or PAI that play a central role in body homeostasis, including the regulation of food intake and energy balance, insulin action, lipid and glucose metabolism, angiogenesis and vascular remodelling, regulation of blood pressure and coagulation. Moreover, downstream UTS2 is located in the TNFRSF9 gene, which encodes a receptor for tumor necrosis factor (TNF), another adipokine. Three polymorphisms within the coding region of TNFRSF9 (namely rs161827, rs161811 and rs519546) have been shown to modulate UTS2 gene expression, as revealed by the use of the mRNA by SNP Browser 1.0.1. (http://www.sph.umich.edu/csg/liang/asthma/) [26], [27]. In our analysis, we have only included two polymorphisms at the TNFRSF9 gene region (rs2453021 and rs863171) that are not associated with any of the traits analysed. These two SNPs are in the same block of UTS2 and the three polymorphisms that modulate UTS2 mRNA levels.

The role of UII in glucose homeostasis is well established. Plasma UII levels have been reported to be almost twice as high in diabetic patients compared with healthy subjects [28]. This increase in UII level does not seem to be a consequence of hyperglycemia, but UII itself may be responsible for hyperglycemia. UII and the UTR are both expressed in the pancreatic islets, where it inhibits insulin release without affecting glucagon or somatostatin levels [6], [7]. A recent report has, however, suggested that inhibition of glucose-induced insulin secretion in beta cells is mediated by the UII receptor and PKC pathway, as well as the somatostatin receptor, which could be activated by high dose of UII [29]. Other proposed mechanisms include activation of L-type Ca^2+^ channels, increase in the phospholipid turnover, activation of the adenylate cyclase/cAMP system (GLP-1) or blockage of ATP-dependent K^+^ channels [30]. In cardiomyocytes, UII also increases phosphorylation of Akt and its downstream target GSK-3β, a serine/threonine protein kinase discovered for its property to inhibit glycogen synthase that has been implicated in many disease states, among others tumorigenesis, diabetes or neurodegenerative diseases [31]. In salmon, UII increases glucose-6-phosphatase activity and reduces liver glycogen content [32].

Activation of the UII system was also proposed to be associated with the development of metabolic syndrome [8]. Although UII doesn't seem to be implicated in the initiation of the individual factors comprising the metabolic syndrome, there is evidence that UII plays a role in the development of each factor [33]. UII plays a role in hypertension, in both dyslipidemia and obesity, and in hyperglycemia [5]. Increased plasma UII levels in patients with T2DM was recently associated with the metabolic syndrome phenotype [34]. The primary clinical outcomes of the metabolic syndrome are cardiovascular diseases, such as coronary heart diseases or stroke. Several reports have shown that plasma UII levels are increased in cardiovascular diseases such as congestive heart failure and other cardiac diseases (for review see Ross et al. 2010 [5]). Expression of both UII and UT receptors are increased in the heart of patients after myocardial infarction, suggesting a possible pathological role in cardiac remodeling [35]. Indeed, UII was shown to be involved in cardiac fibrosis, hypertrophy [35] and remodeling [36]. Interestingly, several studies have linked hyperglycaemia, at the time of hospital admission, with higher mortality in patients with acute coronary syndrome (ACS) [37]–[43]. The effect of admission hyperglycaemia on mortality seems to be independent of a previous diagnosis of diabetes mellitus; indeed, some studies have suggested that mortality may be higher in patients with hyperglycaemia without a previous history of diabetes, compared to those with known history of diabetes [44], [45]. This last study showed that given the same degree of hyperglycemia on admission, known diabetes has a protective influence on short-term outcomes in hospitalized ACS patients. The mechanism by means chronic diabetes mitigates the effect of hyperglycemia in acute cardiovascular event is unknown.

Given that UII has been proven to be a potent insulinostatic peptide, urotensin II receptor antagonists have been proposed as potential drugs for treating the impaired insulin secretion characteristic of T2DM patients. In a follow-up study, Clozel et al. observed that chronic oral treatment with palosuran, a potent and selective UTR antagonist, improved survival, increased insulin, and slowed the increase in glycemia, glycosylated hemoglobin, and serum lipids. Furthermore, palosuran increased renal blood flow and delayed the development of proteinuria and renal damage [46]. Other authors have reported that, in the absence of exogenous UII, palosuran potentiated glucose-induced insulin release in perfused rat pancreas [47] and decreased 24-hour urinary albumin excretion rate in diabetic patients with renal failure with regard to their disease progression [48]. However, other studies have reported that palosuran has no effect on the first-phase insulin response, insulin secretion and blood glucose levels during the meal tolerance test or on homeostasis model assessment-insulin resistance score of diabetic patients in diet-treated patients with T2DM [49]. Furthermore palosuran did not affect albuminuria, blood pressure, glomerular filtration rate, or renal plasma flow significantly in hypertensive patients with type 2 diabetic nephropathy [50].

In conclusion, our results are compatible with a role for UII in glucose homeostasis and diabetes. Although we have not examined previously associated polymorphisms such as S89A, we have studied highly linked SNPs in UTS2 gene region with positive results. However, we cannot rule out the possibility that PER3 gene underlies the reported associations, although the biological relevance of PER3 in glucose metabolism is not well established. Further analysis in independent Caucasian populations and functional analysis are needed to answer this question.

## Methods

### Study design

This study comprises 801 non related Caucasian men (n = 433, 54.05%) and women (n = 368, 45.95%) who were recruited by a simple random sampling approach from a cross-sectional population-based epidemiological survey in Spain, aimed to investigate the prevalence of anthropometric and physiological parameters related to obesity and other components of MS [51], [52]. Participants with previous diagnosis of type 1 diabetes were excluded from the study.

All participants gave their written consent to participate in the study. The study protocol was approved by the Ethics Committee of the Hospital Clínico San Carlos of Madrid.

### Measurements

#### Biochemical determinations

After an overnight fasting period, 20 ml of blood were obtained from an antecubital vein without compression. Plasma glucose was determined in duplicate by a glucose-oxidase method adapted to an Autoanalyzer (Hitachi 704, Boehringer Mannheim, Germany). Serum insulin was determined by RIA (Human Insulin Specific RIA kit, Linco Research Inc., St Louis MO, USA). Oral glucose tolerance test (OGTT) using 75 g of glucose was performed according to the WHO recommendations. Two hours after glucose administration, blood samples were obtained for the determination of glucose levels and interpreted the results according to Genuth et al. [53]. The crude prevalences of T2DM, impaired fasting glucose (IFG) and impaired glucose tolerance (IGT) were respectively 8.7%, 13.6% and 15.0%.

Insulin resistance was estimated by the homeostasis model assessment (HOMA-IR) method according to the formula: Insulin (mU/l)×Glucose (mmol/l)/22.5 [54].

#### Genotypes

Genotypic data were derived from a genome wide scan performed with the 250 k NspIAffymetrix chip. Genotyping was done according to manufacturer's instructions. A 1% of the samples are duplicated in the study, being the overall concordance between them throughout the genome in the range 95–98%. After the application of additional genotyping quality controls (SNPs genotyped in at least 90% of individuals, with minor allele frequency (MAF)≥0.05, and Hardy-Weinberg equilibrium p>0.05), we performed a genome wide imputation using MACH 1 software (http://www.sph.umich.edu/csg/abecasis/MACH) [55] and CEU HapMap phased haplotypic data (http://www.HapMap.org).

### Statistical analysis

From the GWAS data, we extracted the SNPs included in the region from 7,711,426 to 8,168,634 bp (NCBI37/hg19) of chromosome 1 (including the largest UTS2 transcript ±200 kb) (Table S5). A total of 98SNPswere analysed for association with fasting glucose, 120-min glucose (after an OGTT), fasting insulin and HOMA, using the linear regression procedure included in the PLINK software [56]. Phenotypic values were log transformed to achieve approximately normal distribution. All the studies were adjusted by sex, age, BMI, smoking (defined as present or past history of smoking of at least five cigarettes per day for a minimum of 5 years), alcohol consumption (defined as a daily intake of more than 1 drink, equivalent to 10 g of pure ethanol), and physical activity (physical activity was defined as light or moderate activity for a minimum of 60 minutes per session at least three times a week). A p value under 0.05 is interpreted as significant.

For the analysis of linkage disequilibrium (LD) patterns and selection of tagging SNPs we used Haploview software [57].

For haplotypic association analysis, we have also employed PLINK v1.06 software [56]. Only haplotypes with a population frequency over 5% were retained for analysis.

## Acknowledgments

We thank patients involved in this study. We are deeply grateful to Mari Carmen Rivero, Juan Velasco and Ana Salinas for their technical work. We also thank Antonio Gonzalez and Javier Gayán for statistical advice and Juliana Martínez for the review of the manuscript.

## Supporting Information

Table S1 **Fasting glucose: genetic association analysis at** ***UTS2*** **gene region.**
(DOC)

Table S2 **OGTT: genetic association analysis at** ***UTS2*** **gene region.**
(DOC)

Table S3 **Fasting insulin: genetic association analysis at** ***UTS2*** **gene region.**
(DOC)

Table S4 **HOMA: genetic association analysis at** ***UTS2*** **gene region.**
(DOC)

Table S5 **Genotyped polymorphisms included in the Affymetrix 250 k NspI chip.**
(DOC)
