# Supplementary material for: Association Analysis of Urotensin II Gene (UTS2) and Flanking Regions with Biochemical Parameters Related to Insulin Resistance
Source: PLoS One. 2011 Apr 29;6(4):e19327. doi: 10.1371/journal.pone.0019327 (PMC3084835; doi:10.1371/journal.pone.0019327)
Supplement: Table S1 — Fasting glucose: genetic association analysis at UTS2 gene region. (DOC) [file pone.0019327.s001.doc]

Table S1. Fasting glucose: genetic association analysis at *UTS2* gene region.

| **GENE** | **SNP** | **Bp (hg19)** | **A1** | **BETA** | **SE** | **L95** | **U95** | **STAT** | **P** |
| --- | --- | --- | --- | --- | --- | --- | --- | --- | --- |
| CAMTA1 | rs4908665 | 7,715,776 | T | 0.012 | 0.010 | -0.009 | 0.032 | 1.117 | 0.264 |
| CAMTA1 | rs9434881 | 7,716,768 | C | 0.012 | 0.010 | -0.009 | 0.032 | 1.117 | 0.264 |
| CAMTA1 | rs17031253 | 7,717,080 | A | 0.006 | 0.011 | -0.017 | 0.028 | 0.498 | 0.619 |
| CAMTA1 | rs9434882 | 7,717,962 | C | 0.012 | 0.010 | -0.009 | 0.032 | 1.117 | 0.264 |
| CAMTA1 | rs17376559 | 7,718,093 | A | 0.006 | 0.011 | -0.017 | 0.028 | 0.498 | 0.619 |
| CAMTA1 | rs17031274 | 7,719,653 | G | 0.006 | 0.011 | -0.017 | 0.028 | 0.498 | 0.619 |
| CAMTA1 | rs12071290 | 7,719,695 | C | 0.006 | 0.011 | -0.016 | 0.029 | 0.557 | 0.577 |
| CAMTA1 | rs2995026 | 7,777,415 | T | 0.033 | 0.016 | 0.002 | 0.064 | 2.119 | **0.034** |
| CAMTA1 | rs6693805 | 7,784,438 | A | -0.020 | 0.013 | -0.047 | 0.006 | -1.532 | 0.126 |
| CAMTA1 | rs4908688 | 7,795,554 | T | -0.020 | 0.013 | -0.047 | 0.006 | -1.532 | 0.126 |
| CAMTA1 | rs6577456 | 7,818,679 | G | -0.025 | 0.010 | -0.044 | -0.005 | -2.494 | **0.013** |
| CAMTA1 | rs697672 | 7,826,347 | C | 0.022 | 0.014 | -0.005 | 0.049 | 1.607 | 0.108 |
| CAMTA1 | rs41454244 | 7,829,286 | C | 0.008 | 0.015 | -0.021 | 0.038 | 0.572 | 0.568 |
| VAMP3 | rs697674 | 7,837,878 | G | -0.020 | 0.013 | -0.047 | 0.006 | -1.532 | 0.126 |
| VAMP3 | rs697675 | 7,838,113 | C | -0.020 | 0.013 | -0.047 | 0.006 | -1.532 | 0.126 |
| PER3 | rs836755 | 7,846,527 | C | -0.024 | 0.010 | -0.044 | -0.004 | -2.306 | **0.021** |
| PER3 | rs228727 | 7,847,836 | C | -0.025 | 0.010 | -0.044 | -0.005 | -2.494 | **0.013** |
| PER3 | rs707463 | 7,850,062 | T | -0.024 | 0.010 | -0.044 | -0.004 | -2.306 | **0.021** |
| PER3 | rs697686 | 7,850,218 | T | -0.024 | 0.010 | -0.044 | -0.004 | -2.306 | **0.021** |
| PER3 | rs4908694 | 7,850,898 | T | -0.020 | 0.013 | -0.047 | 0.006 | -1.532 | 0.126 |
| PER3 | rs696306 | 7,854,998 | T | -0.023 | 0.010 | -0.043 | -0.003 | -2.238 | **0.026** |
| PER3 | rs1012477 | 7,858,135 | C | -0.020 | 0.013 | -0.047 | 0.006 | -1.532 | 0.126 |
| PER3 | rs707465 | 7,861,304 | C | -0.023 | 0.010 | -0.043 | -0.003 | -2.238 | **0.026** |
| PER3 | rs228641 | 7,862,899 | T | -0.006 | 0.028 | -0.061 | 0.049 | -0.218 | 0.827 |
| PER3 | rs10864316 | 7,872,076 | G | -0.005 | 0.013 | -0.032 | 0.021 | -0.412 | 0.681 |
| PER3 | rs4908482 | 7,877,488 | A | -0.025 | 0.010 | -0.044 | -0.005 | -2.494 | **0.013** |
| PER3 | rs10746473 | 7,878,056 | A | -0.025 | 0.010 | -0.044 | -0.005 | -2.494 | **0.013** |
| PER3 | rs12141033 | 7,878,547 | A | -0.025 | 0.010 | -0.044 | -0.005 | -2.494 | **0.013** |
| PER3 | rs228688 | 7,879,130 | T | -0.025 | 0.010 | -0.044 | -0.005 | -2.494 | **0.013** |
| PER3 | rs10462018 | 7,879,627 | T | -0.020 | 0.013 | -0.047 | 0.006 | -1.532 | 0.126 |
| PER3 | rs228691 | 7,880,469 | A | -0.025 | 0.010 | -0.044 | -0.005 | -2.494 | **0.013** |
| PER3 | rs10462020 | 7,880,683 | G | -0.005 | 0.013 | -0.032 | 0.021 | -0.412 | 0.681 |
| PER3 | rs17374292 | 7,881,234 | T | -0.020 | 0.013 | -0.047 | 0.006 | -1.532 | 0.126 |
| PER3 | rs228694 | 7,883,834 | A | -0.025 | 0.010 | -0.044 | -0.005 | -2.494 | **0.013** |
| PER3 | rs697690 | 7,884,580 | C | -0.024 | 0.010 | -0.044 | -0.004 | -2.308 | **0.021** |
| PER3 | rs17374439 | 7,888,438 | T | -0.004 | 0.013 | -0.030 | 0.021 | -0.330 | 0.742 |
| PER3 | rs12061787 | 7,888,730 | C | -0.026 | 0.014 | -0.053 | 0.001 | -1.875 | 0.061 |
| PER3 | rs228664 | 7,891,083 | A | 0.005 | 0.030 | -0.054 | 0.063 | 0.153 | 0.879 |
| PER3 | rs12130462 | 7,891,378 | T | -0.005 | 0.013 | -0.032 | 0.021 | -0.412 | 0.681 |
| PER3 | rs10462021 | 7,897,133 | G | -0.005 | 0.013 | -0.032 | 0.021 | -0.412 | 0.681 |
| PER3 | rs12741937 | 7,897,622 | T | -0.024 | 0.014 | -0.051 | 0.002 | -1.788 | 0.074 |
| UTS2 | rs228652 | 7,908,888 | A | 0.018 | 0.011 | -0.002 | 0.039 | 1.726 | 0.085 |
| UTS2 | rs4908486 | 7,914,835 | T | -0.018 | 0.010 | -0.038 | 0.002 | -1.812 | 0.070 |
| UTS2 | rs228637 | 7,917,632 | A | 0.019 | 0.013 | -0.006 | 0.044 | 1.522 | 0.128 |
| UTS2 | rs17374781 | 7,919,363 | C | -0.027 | 0.013 | -0.052 | -0.002 | -2.157 | **0.031** |
| UTS2 | rs531485 | 7,921,952 | G | 0.012 | 0.012 | -0.011 | 0.035 | 1.035 | 0.301 |
| UTS2 | rs515830 | 7,923,586 | A | 0.010 | 0.012 | -0.014 | 0.033 | 0.818 | 0.414 |
| UTS2 | rs504560 | 7,926,542 | A | -0.020 | 0.011 | -0.041 | 0.001 | -1.854 | 0.064 |
| UTS2 | rs500508 | 7,927,456 | T | -0.020 | 0.011 | -0.041 | 0.001 | -1.881 | 0.060 |
| UTS2 | rs579992 | 7,927,981 | C | 0.003 | 0.018 | -0.032 | 0.037 | 0.162 | 0.871 |
| UTS2 | rs2066980 | 7,928,181 | G | 0.010 | 0.011 | -0.011 | 0.031 | 0.946 | 0.344 |
| UTS2 | rs2066978 | 7,928,759 | C | 0.020 | 0.011 | -0.002 | 0.043 | 1.768 | 0.077 |
| UTS2 | rs228725 | 7,929,819 | T | 0.017 | 0.010 | -0.003 | 0.036 | 1.681 | 0.093 |
| UTS2 | rs228724 | 7,930,554 | C | 0.017 | 0.010 | -0.003 | 0.036 | 1.681 | 0.093 |
| UTS2 | rs228721 | 7,931,588 | A | 0.011 | 0.012 | -0.012 | 0.033 | 0.930 | 0.353 |
| UTS2 | rs228720 | 7,933,457 | G | 0.017 | 0.010 | -0.003 | 0.036 | 1.681 | 0.093 |
| UTS2 | rs228719 | 7,934,171 | A | 0.017 | 0.010 | -0.003 | 0.036 | 1.681 | 0.093 |
| UTS2 | rs228716 | 7,936,272 | G | 0.017 | 0.010 | -0.003 | 0.036 | 1.681 | 0.093 |
| UTS2 | rs228714 | 7,938,648 | G | 0.017 | 0.010 | -0.003 | 0.036 | 1.681 | 0.093 |
| UTS2 | rs228703 | 7,944,264 | G | 0.000 | 0.009 | -0.018 | 0.018 | 0.009 | 0.993 |
| UTS2 | rs1040396 | 7,952,404 | C | -0.005 | 0.010 | -0.025 | 0.014 | -0.543 | 0.588 |
| UTS2 | rs1040397 | 7,952,427 | A | -0.005 | 0.010 | -0.025 | 0.014 | -0.543 | 0.588 |
| UTS2 | rs665244 | 7,970,248 | A | 0.046 | 0.018 | 0.010 | 0.081 | 2.526 | **0.012** |
| TNFRSF9 | rs2453021 | 7,989,566 | T | 0.009 | 0.010 | -0.011 | 0.029 | 0.866 | 0.387 |
| TNFRSF9 | rs863171 | 7,992,615 | T | 0.012 | 0.010 | -0.008 | 0.031 | 1.197 | 0.232 |
|  | rs2493215 | 8,007,716 | G | 0.016 | 0.010 | -0.003 | 0.034 | 1.605 | 0.109 |
|  | rs226474 | 8,009,763 | T | 0.016 | 0.010 | -0.003 | 0.034 | 1.605 | 0.109 |
| PARK7 | rs226249 | 8,021,778 | C | 0.010 | 0.010 | -0.009 | 0.029 | 1.022 | 0.307 |
| PARK7 | rs3766606 | 8,022,197 | T | -0.009 | 0.013 | -0.034 | 0.016 | -0.745 | 0.457 |
| PARK7 | rs226251 | 8,024,690 | T | 0.010 | 0.010 | -0.009 | 0.029 | 1.022 | 0.307 |
| PARK7 | rs7517357 | 8,025,275 | T | -0.009 | 0.013 | -0.034 | 0.016 | -0.745 | 0.457 |
| PARK7 | rs161802 | 8,042,826 | T | -0.010 | 0.013 | -0.035 | 0.015 | -0.777 | 0.437 |
| PARK7 | rs225119 | 8,044,361 | T | 0.010 | 0.010 | -0.009 | 0.029 | 1.048 | 0.295 |
|  | rs12727642 | 8,046,672 | A | -0.010 | 0.013 | -0.035 | 0.015 | -0.777 | 0.437 |
|  | rs17367289 | 8,053,135 | G | -0.010 | 0.013 | -0.035 | 0.015 | -0.777 | 0.437 |
|  | rs225100 | 8,066,914 | T | 0.010 | 0.010 | -0.009 | 0.029 | 1.048 | 0.295 |
| ERRFI1 | rs397349 | 8,074,872 | C | -0.014 | 0.012 | -0.038 | 0.010 | -1.124 | 0.262 |
| ERRFI1 | rs400736 | 8,078,309 | T | 0.010 | 0.010 | -0.009 | 0.029 | 1.048 | 0.295 |
| ERRFI1 | rs10489450 | 8,079,301 | T | -0.014 | 0.012 | -0.038 | 0.010 | -1.124 | 0.262 |
| ERRFI1 | rs442862 | 8,079,494 | T | 0.010 | 0.010 | -0.009 | 0.029 | 1.048 | 0.295 |
| ERRFI1 | rs28624 | 8,084,355 | C | -0.014 | 0.012 | -0.038 | 0.010 | -1.124 | 0.262 |
| ERRFI1 | rs408320 | 8,085,328 | T | 0.010 | 0.010 | -0.009 | 0.029 | 1.048 | 0.295 |
|  | rs225132 | 8,095,500 | G | -0.014 | 0.012 | -0.038 | 0.010 | -1.124 | 0.262 |
|  | rs6577459 | 8,100,173 | T | -0.019 | 0.014 | -0.046 | 0.009 | -1.325 | 0.186 |
|  | rs1883679 | 8,100,451 | G | -0.014 | 0.012 | -0.038 | 0.010 | -1.124 | 0.262 |
|  | rs2050198 | 8,111,839 | G | -0.014 | 0.012 | -0.038 | 0.010 | -1.124 | 0.262 |
|  | rs12753070 | 8,114,319 | G | -0.017 | 0.013 | -0.043 | 0.008 | -1.318 | 0.188 |
|  | rs4908724 | 8,119,251 | T | -0.017 | 0.014 | -0.044 | 0.010 | -1.226 | 0.220 |
|  | rs12748993 | 8,129,507 | G | -0.014 | 0.012 | -0.038 | 0.010 | -1.124 | 0.262 |
|  | rs12730860 | 8,132,462 | C | -0.014 | 0.012 | -0.038 | 0.010 | -1.124 | 0.262 |
|  | rs7539255 | 8,133,352 | C | -0.014 | 0.012 | -0.038 | 0.010 | -1.124 | 0.262 |
|  | rs12736494 | 8,136,016 | A | -0.014 | 0.012 | -0.038 | 0.010 | -1.124 | 0.262 |
|  | rs12758337 | 8,145,294 | T | 0.019 | 0.010 | -0.002 | 0.039 | 1.802 | 0.072 |
|  | rs11121086 | 8,151,224 | A | 0.019 | 0.010 | -0.002 | 0.039 | 1.802 | 0.072 |
|  | rs7553544 | 8,165,719 | A | 0.018 | 0.010 | -0.003 | 0.038 | 1.693 | 0.091 |
|  | rs10864330 | 8,168,564 | T | 0.018 | 0.010 | -0.003 | 0.038 | 1.693 | 0.091 |
|  | rs11121090 | 8,168,634 | T | 0.018 | 0.010 | -0.003 | 0.038 | 1.693 | 0.091 |
