# Supplementary material for: Association Analysis of Urotensin II Gene (UTS2) and Flanking Regions with Biochemical Parameters Related to Insulin Resistance
Source: PLoS One. 2011 Apr 29;6(4):e19327. doi: 10.1371/journal.pone.0019327 (PMC3084835; doi:10.1371/journal.pone.0019327)
Supplement: Table S2 — OGTT: genetic association analysis at UTS2 gene region. (DOC) [file pone.0019327.s002.doc]

Table S2. OGTT: genetic association analysis at *UTS2* gene region.

| **GENE** | **SNP** | **Bp (hg19)** | **A1** | **BETA** | **SE** | **L95** | **U95** | **STAT** | **P** |
| --- | --- | --- | --- | --- | --- | --- | --- | --- | --- |
| CAMTA1 | rs4908665 | 7,715,776 | T | 0.002 | 0.021 | -0.040 | 0.043 | 0.077 | 0.939 |
| CAMTA1 | rs9434881 | 7,716,768 | C | 0.002 | 0.021 | -0.040 | 0.043 | 0.077 | 0.939 |
| CAMTA1 | rs17031253 | 7,717,080 | A | -0.009 | 0.023 | -0.054 | 0.036 | -0.383 | 0.702 |
| CAMTA1 | rs9434882 | 7,717,962 | C | 0.002 | 0.021 | -0.040 | 0.043 | 0.077 | 0.939 |
| CAMTA1 | rs17376559 | 7,718,093 | A | -0.009 | 0.023 | -0.054 | 0.036 | -0.383 | 0.702 |
| CAMTA1 | rs17031274 | 7,719,653 | G | -0.009 | 0.023 | -0.054 | 0.036 | -0.383 | 0.702 |
| CAMTA1 | rs12071290 | 7,719,695 | C | -0.007 | 0.023 | -0.052 | 0.039 | -0.285 | 0.776 |
| CAMTA1 | rs2995026 | 7,777,415 | T | 0.073 | 0.033 | 0.008 | 0.138 | 2.193 | **0.029** |
| CAMTA1 | rs6693805 | 7,784,438 | A | -0.005 | 0.027 | -0.057 | 0.047 | -0.182 | 0.856 |
| CAMTA1 | rs4908688 | 7,795,554 | T | -0.005 | 0.027 | -0.057 | 0.047 | -0.182 | 0.856 |
| CAMTA1 | rs6577456 | 7,818,679 | G | -0.005 | 0.020 | -0.044 | 0.035 | -0.236 | 0.814 |
| CAMTA1 | rs697672 | 7,826,347 | C | 0.032 | 0.028 | -0.024 | 0.087 | 1.115 | 0.265 |
| CAMTA1 | rs41454244 | 7,829,286 | C | 0.021 | 0.030 | -0.039 | 0.080 | 0.678 | 0.498 |
| VAMP3 | rs697674 | 7,837,878 | G | -0.005 | 0.027 | -0.057 | 0.047 | -0.182 | 0.856 |
| VAMP3 | rs697675 | 7,838,113 | C | -0.005 | 0.027 | -0.057 | 0.047 | -0.182 | 0.856 |
| PER3 | rs836755 | 7,846,527 | C | 0.001 | 0.021 | -0.040 | 0.042 | 0.058 | 0.954 |
| PER3 | rs228727 | 7,847,836 | C | -0.005 | 0.020 | -0.044 | 0.035 | -0.236 | 0.814 |
| PER3 | rs707463 | 7,850,062 | T | 0.001 | 0.021 | -0.040 | 0.042 | 0.058 | 0.954 |
| PER3 | rs697686 | 7,850,218 | T | 0.001 | 0.021 | -0.040 | 0.042 | 0.058 | 0.954 |
| PER3 | rs4908694 | 7,850,898 | T | -0.005 | 0.027 | -0.057 | 0.047 | -0.182 | 0.856 |
| PER3 | rs696306 | 7,854,998 | T | 0.011 | 0.021 | -0.030 | 0.052 | 0.514 | 0.608 |
| PER3 | rs1012477 | 7,858,135 | C | -0.005 | 0.027 | -0.057 | 0.047 | -0.182 | 0.856 |
| PER3 | rs707465 | 7,861,304 | C | 0.011 | 0.021 | -0.030 | 0.052 | 0.514 | 0.608 |
| PER3 | rs228641 | 7,862,899 | T | -0.044 | 0.058 | -0.158 | 0.069 | -0.765 | 0.445 |
| PER3 | rs10864316 | 7,872,076 | G | -0.006 | 0.028 | -0.060 | 0.048 | -0.214 | 0.831 |
| PER3 | rs4908482 | 7,877,488 | A | -0.005 | 0.020 | -0.044 | 0.035 | -0.236 | 0.814 |
| PER3 | rs10746473 | 7,878,056 | A | -0.005 | 0.020 | -0.044 | 0.035 | -0.236 | 0.814 |
| PER3 | rs12141033 | 7,878,547 | A | -0.005 | 0.020 | -0.044 | 0.035 | -0.236 | 0.814 |
| PER3 | rs228688 | 7,879,130 | T | -0.005 | 0.020 | -0.044 | 0.035 | -0.236 | 0.814 |
| PER3 | rs10462018 | 7,879,627 | T | -0.005 | 0.027 | -0.057 | 0.047 | -0.182 | 0.856 |
| PER3 | rs228691 | 7,880,469 | A | -0.005 | 0.020 | -0.044 | 0.035 | -0.236 | 0.814 |
| PER3 | rs10462020 | 7,880,683 | G | -0.006 | 0.028 | -0.060 | 0.048 | -0.214 | 0.831 |
| PER3 | rs17374292 | 7,881,234 | T | -0.005 | 0.027 | -0.057 | 0.047 | -0.182 | 0.856 |
| PER3 | rs228694 | 7,883,834 | A | -0.005 | 0.020 | -0.044 | 0.035 | -0.236 | 0.814 |
| PER3 | rs697690 | 7,884,580 | C | 0.011 | 0.021 | -0.031 | 0.052 | 0.505 | 0.614 |
| PER3 | rs17374439 | 7,888,438 | T | -0.006 | 0.027 | -0.059 | 0.047 | -0.229 | 0.819 |
| PER3 | rs12061787 | 7,888,730 | C | -0.015 | 0.027 | -0.068 | 0.039 | -0.535 | 0.593 |
| PER3 | rs228664 | 7,891,083 | A | -0.035 | 0.062 | -0.158 | 0.087 | -0.566 | 0.572 |
| PER3 | rs12130462 | 7,891,378 | T | -0.006 | 0.028 | -0.060 | 0.048 | -0.214 | 0.831 |
| PER3 | rs10462021 | 7,897,133 | G | -0.006 | 0.028 | -0.060 | 0.048 | -0.214 | 0.831 |
| PER3 | rs12741937 | 7,897,622 | T | -0.004 | 0.027 | -0.057 | 0.049 | -0.154 | 0.878 |
| UTS2 | rs228652 | 7,908,888 | A | 0.023 | 0.022 | -0.021 | 0.066 | 1.006 | 0.315 |
| UTS2 | rs4908486 | 7,914,835 | T | -0.002 | 0.021 | -0.043 | 0.039 | -0.102 | 0.919 |
| UTS2 | rs228637 | 7,917,632 | A | 0.070 | 0.027 | 0.016 | 0.123 | 2.550 | **0.011** |
| UTS2 | rs17374781 | 7,919,363 | C | -0.013 | 0.025 | -0.063 | 0.037 | -0.520 | 0.603 |
| UTS2 | rs531485 | 7,921,952 | G | 0.053 | 0.025 | 0.005 | 0.102 | 2.144 | **0.032** |
| UTS2 | rs515830 | 7,923,586 | A | 0.058 | 0.025 | 0.009 | 0.108 | 2.323 | **0.021** |
| UTS2 | rs504560 | 7,926,542 | A | -0.046 | 0.022 | -0.089 | -0.003 | -2.079 | **0.038** |
| UTS2 | rs500508 | 7,927,456 | T | -0.047 | 0.022 | -0.090 | -0.004 | -2.150 | **0.032** |
| UTS2 | rs579992 | 7,927,981 | C | -0.058 | 0.036 | -0.128 | 0.012 | -1.627 | 0.104 |
| UTS2 | rs2066980 | 7,928,181 | G | 0.024 | 0.022 | -0.019 | 0.068 | 1.083 | 0.279 |
| UTS2 | rs2066978 | 7,928,759 | C | 0.010 | 0.024 | -0.036 | 0.057 | 0.433 | 0.665 |
| UTS2 | rs228725 | 7,929,819 | T | -0.023 | 0.020 | -0.063 | 0.017 | -1.107 | 0.269 |
| UTS2 | rs228724 | 7,930,554 | C | -0.023 | 0.020 | -0.063 | 0.017 | -1.107 | 0.269 |
| UTS2 | rs228721 | 7,931,588 | A | 0.058 | 0.025 | 0.009 | 0.106 | 2.337 | **0.020** |
| UTS2 | rs228720 | 7,933,457 | G | -0.023 | 0.020 | -0.063 | 0.017 | -1.107 | 0.269 |
| UTS2 | rs228719 | 7,934,171 | A | -0.023 | 0.020 | -0.063 | 0.017 | -1.107 | 0.269 |
| UTS2 | rs228716 | 7,936,272 | G | -0.023 | 0.020 | -0.063 | 0.017 | -1.107 | 0.269 |
| UTS2 | rs228714 | 7,938,648 | G | -0.023 | 0.020 | -0.063 | 0.017 | -1.107 | 0.269 |
| UTS2 | rs228703 | 7,944,264 | G | 0.034 | 0.019 | -0.004 | 0.072 | 1.737 | 0.083 |
| UTS2 | rs1040396 | 7,952,404 | C | 0.020 | 0.021 | -0.020 | 0.061 | 0.978 | 0.328 |
| UTS2 | rs1040397 | 7,952,427 | A | 0.020 | 0.021 | -0.020 | 0.061 | 0.978 | 0.328 |
| UTS2 | rs665244 | 7,970,248 | A | 0.089 | 0.039 | 0.012 | 0.166 | 2.258 | **0.024** |
| TNFRSF9 | rs2453021 | 7,989,566 | T | 0.004 | 0.021 | -0.037 | 0.045 | 0.181 | 0.856 |
| TNFRSF9 | rs863171 | 7,992,615 | T | 0.002 | 0.021 | -0.038 | 0.043 | 0.117 | 0.907 |
|  | rs2493215 | 8,007,716 | G | 0.011 | 0.020 | -0.028 | 0.051 | 0.548 | 0.584 |
|  | rs226474 | 8,009,763 | T | 0.011 | 0.020 | -0.028 | 0.051 | 0.548 | 0.584 |
| PARK7 | rs226249 | 8,021,778 | C | 0.008 | 0.021 | -0.033 | 0.048 | 0.366 | 0.715 |
| PARK7 | rs3766606 | 8,022,197 | T | -0.011 | 0.026 | -0.062 | 0.039 | -0.437 | 0.662 |
| PARK7 | rs226251 | 8,024,690 | T | 0.008 | 0.021 | -0.033 | 0.048 | 0.366 | 0.715 |
| PARK7 | rs7517357 | 8,025,275 | T | -0.011 | 0.026 | -0.062 | 0.039 | -0.437 | 0.662 |
| PARK7 | rs161802 | 8,042,826 | T | -0.006 | 0.026 | -0.056 | 0.044 | -0.235 | 0.815 |
| PARK7 | rs225119 | 8,044,361 | T | -0.002 | 0.020 | -0.040 | 0.037 | -0.075 | 0.940 |
|  | rs12727642 | 8,046,672 | A | -0.006 | 0.026 | -0.056 | 0.044 | -0.235 | 0.815 |
|  | rs17367289 | 8,053,135 | G | -0.006 | 0.026 | -0.056 | 0.044 | -0.235 | 0.815 |
|  | rs225100 | 8,066,914 | T | -0.001 | 0.020 | -0.040 | 0.038 | -0.070 | 0.944 |
| ERRFI1 | rs397349 | 8,074,872 | C | -0.005 | 0.025 | -0.055 | 0.044 | -0.205 | 0.838 |
| ERRFI1 | rs400736 | 8,078,309 | T | -0.001 | 0.020 | -0.040 | 0.038 | -0.070 | 0.944 |
| ERRFI1 | rs10489450 | 8,079,301 | T | -0.005 | 0.025 | -0.055 | 0.044 | -0.205 | 0.838 |
| ERRFI1 | rs442862 | 8,079,494 | T | -0.001 | 0.020 | -0.040 | 0.038 | -0.070 | 0.944 |
| ERRFI1 | rs28624 | 8,084,355 | C | -0.005 | 0.025 | -0.055 | 0.044 | -0.205 | 0.838 |
| ERRFI1 | rs408320 | 8,085,328 | T | -0.001 | 0.020 | -0.040 | 0.038 | -0.070 | 0.944 |
|  | rs225132 | 8,095,500 | G | -0.005 | 0.025 | -0.055 | 0.044 | -0.205 | 0.838 |
|  | rs6577459 | 8,100,173 | T | -0.003 | 0.028 | -0.058 | 0.052 | -0.110 | 0.912 |
|  | rs1883679 | 8,100,451 | G | -0.005 | 0.025 | -0.055 | 0.044 | -0.205 | 0.838 |
|  | rs2050198 | 8,111,839 | G | -0.005 | 0.025 | -0.055 | 0.044 | -0.205 | 0.838 |
|  | rs12753070 | 8,114,319 | G | -0.008 | 0.027 | -0.060 | 0.044 | -0.314 | 0.754 |
|  | rs4908724 | 8,119,251 | T | -0.004 | 0.028 | -0.059 | 0.051 | -0.136 | 0.892 |
|  | rs12748993 | 8,129,507 | G | -0.005 | 0.025 | -0.055 | 0.044 | -0.205 | 0.838 |
|  | rs12730860 | 8,132,462 | C | -0.005 | 0.025 | -0.055 | 0.044 | -0.205 | 0.838 |
|  | rs7539255 | 8,133,352 | C | -0.005 | 0.025 | -0.055 | 0.044 | -0.205 | 0.838 |
|  | rs12736494 | 8,136,016 | A | -0.005 | 0.025 | -0.055 | 0.044 | -0.205 | 0.838 |
|  | rs12758337 | 8,145,294 | T | 0.015 | 0.021 | -0.027 | 0.057 | 0.706 | 0.481 |
|  | rs11121086 | 8,151,224 | A | 0.015 | 0.021 | -0.027 | 0.057 | 0.706 | 0.481 |
|  | rs7553544 | 8,165,719 | A | 0.017 | 0.021 | -0.025 | 0.059 | 0.790 | 0.430 |
|  | rs10864330 | 8,168,564 | T | 0.017 | 0.021 | -0.025 | 0.059 | 0.790 | 0.430 |
|  | rs11121090 | 8,168,634 | T | 0.017 | 0.021 | -0.025 | 0.059 | 0.790 | 0.430 |
