# Supplementary material for: Association Analysis of Urotensin II Gene (UTS2) and Flanking Regions with Biochemical Parameters Related to Insulin Resistance
Source: PLoS One. 2011 Apr 29;6(4):e19327. doi: 10.1371/journal.pone.0019327 (PMC3084835; doi:10.1371/journal.pone.0019327)
Supplement: Table S3 — Fasting insulin: genetic association analysis at UTS2 gene region. (DOC) [file pone.0019327.s003.doc]

Table S3. Fasting insulin: genetic association analysis at *UTS2* gene region.

| **GENE** | **SNP** | **Bp (hg19)** | **A1** | **BETA** | **SE** | **L95** | | **U95** | | **STAT** | | | **P** |
| --- | --- | --- | --- | --- | --- | --- | --- | --- | --- | --- | --- | --- | --- |
| CAMTA1 | rs4908665 | 7,715,776 | T | -0.012 | 0.033 | | -0.078 | | 0.053 | | -0.364 | 0.716 | |
| CAMTA1 | rs9434881 | 7,716,768 | C | -0.012 | 0.033 | | -0.078 | | 0.053 | | -0.364 | 0.716 | |
| CAMTA1 | rs17031253 | 7,717,080 | A | -0.029 | 0.036 | | -0.100 | | 0.041 | | -0.815 | 0.416 | |
| CAMTA1 | rs9434882 | 7,717,962 | C | -0.012 | 0.033 | | -0.078 | | 0.053 | | -0.364 | 0.716 | |
| CAMTA1 | rs17376559 | 7,718,093 | A | -0.029 | 0.036 | | -0.100 | | 0.041 | | -0.815 | 0.416 | |
| CAMTA1 | rs17031274 | 7,719,653 | G | -0.029 | 0.036 | | -0.100 | | 0.041 | | -0.815 | 0.416 | |
| CAMTA1 | rs12071290 | 7,719,695 | C | -0.029 | 0.036 | | -0.099 | | 0.042 | | -0.790 | 0.430 | |
| CAMTA1 | rs2995026 | 7,777,415 | T | 0.020 | 0.051 | | -0.080 | | 0.120 | | 0.390 | 0.697 | |
| CAMTA1 | rs6693805 | 7,784,438 | A | -0.062 | 0.043 | | -0.146 | | 0.021 | | -1.461 | 0.144 | |
| CAMTA1 | rs4908688 | 7,795,554 | T | -0.062 | 0.043 | | -0.146 | | 0.021 | | -1.461 | 0.144 | |
| CAMTA1 | rs6577456 | 7,818,679 | G | -0.059 | 0.032 | | -0.121 | | 0.004 | | -1.845 | 0.065 | |
| CAMTA1 | rs697672 | 7,826,347 | C | -0.010 | 0.044 | | -0.095 | | 0.076 | | -0.225 | 0.822 | |
| CAMTA1 | rs41454244 | 7,829,286 | C | 0.017 | 0.047 | | -0.075 | | 0.110 | | 0.362 | 0.717 | |
| VAMP3 | rs697674 | 7,837,878 | G | -0.062 | 0.043 | | -0.146 | | 0.021 | | -1.461 | 0.144 | |
| VAMP3 | rs697675 | 7,838,113 | C | -0.062 | 0.043 | | -0.146 | | 0.021 | | -1.461 | 0.144 | |
| PER3 | rs836755 | 7,846,527 | C | -0.067 | 0.033 | | -0.131 | | -0.003 | | -2.042 | **0.042** | |
| PER3 | rs228727 | 7,847,836 | C | -0.059 | 0.032 | | -0.121 | | 0.004 | | -1.845 | 0.065 | |
| PER3 | rs707463 | 7,850,062 | T | -0.067 | 0.033 | | -0.131 | | -0.003 | | -2.042 | **0.042** | |
| PER3 | rs697686 | 7,850,218 | T | -0.067 | 0.033 | | -0.131 | | -0.003 | | -2.042 | **0.042** | |
| PER3 | rs4908694 | 7,850,898 | T | -0.062 | 0.043 | | -0.146 | | 0.021 | | -1.461 | 0.144 | |
| PER3 | rs696306 | 7,854,998 | T | -0.064 | 0.033 | | -0.129 | | 0.001 | | -1.936 | 0.053 | |
| PER3 | rs1012477 | 7,858,135 | C | -0.062 | 0.043 | | -0.146 | | 0.021 | | -1.461 | 0.144 | |
| PER3 | rs707465 | 7,861,304 | C | -0.064 | 0.033 | | -0.129 | | 0.001 | | -1.936 | 0.053 | |
| PER3 | rs228641 | 7,862,899 | T | 0.036 | 0.089 | | -0.138 | | 0.211 | | 0.408 | 0.684 | |
| PER3 | rs10864316 | 7,872,076 | G | 0.070 | 0.042 | | -0.013 | | 0.153 | | 1.652 | 0.099 | |
| PER3 | rs4908482 | 7,877,488 | A | -0.059 | 0.032 | | -0.121 | | 0.004 | | -1.845 | 0.065 | |
| PER3 | rs10746473 | 7,878,056 | A | -0.059 | 0.032 | | -0.121 | | 0.004 | | -1.845 | 0.065 | |
| PER3 | rs12141033 | 7,878,547 | A | -0.059 | 0.032 | | -0.121 | | 0.004 | | -1.845 | 0.065 | |
| PER3 | rs228688 | 7,879,130 | T | -0.059 | 0.032 | | -0.121 | | 0.004 | | -1.845 | 0.065 | |
| PER3 | rs10462018 | 7,879,627 | T | -0.062 | 0.043 | | -0.146 | | 0.021 | | -1.461 | 0.144 | |
| PER3 | rs228691 | 7,880,469 | A | -0.059 | 0.032 | | -0.121 | | 0.004 | | -1.845 | 0.065 | |
| PER3 | rs10462020 | 7,880,683 | G | 0.070 | 0.042 | | -0.013 | | 0.153 | | 1.652 | 0.099 | |
| PER3 | rs17374292 | 7,881,234 | T | -0.062 | 0.043 | | -0.146 | | 0.021 | | -1.461 | 0.144 | |
| PER3 | rs228694 | 7,883,834 | A | -0.059 | 0.032 | | -0.121 | | 0.004 | | -1.845 | 0.065 | |
| PER3 | rs697690 | 7,884,580 | C | -0.065 | 0.033 | | -0.130 | | 0.000 | | -1.969 | **0.049** | |
| PER3 | rs17374439 | 7,888,438 | T | 0.065 | 0.041 | | -0.016 | | 0.146 | | 1.581 | 0.114 | |
| PER3 | rs12061787 | 7,888,730 | C | -0.075 | 0.044 | | -0.162 | | 0.011 | | -1.707 | 0.088 | |
| PER3 | rs228664 | 7,891,083 | A | 0.074 | 0.095 | | -0.113 | | 0.261 | | 0.778 | 0.437 | |
| PER3 | rs12130462 | 7,891,378 | T | 0.070 | 0.042 | | -0.013 | | 0.153 | | 1.652 | 0.099 | |
| PER3 | rs10462021 | 7,897,133 | G | 0.070 | 0.042 | | -0.013 | | 0.153 | | 1.652 | 0.099 | |
| PER3 | rs12741937 | 7,897,622 | T | -0.067 | 0.044 | | -0.153 | | 0.018 | | -1.546 | 0.123 | |
| UTS2 | rs228652 | 7,908,888 | A | 0.038 | 0.034 | | -0.028 | | 0.105 | | 1.136 | 0.256 | |
| UTS2 | rs4908486 | 7,914,835 | T | 0.005 | 0.033 | | -0.058 | | 0.069 | | 0.169 | 0.866 | |
| UTS2 | rs228637 | 7,917,632 | A | 0.028 | 0.042 | | -0.054 | | 0.110 | | 0.662 | 0.508 | |
| UTS2 | rs17374781 | 7,919,363 | C | -0.046 | 0.041 | | -0.126 | | 0.034 | | -1.126 | 0.260 | |
| UTS2 | rs531485 | 7,921,952 | G | 0.026 | 0.037 | | -0.047 | | 0.099 | | 0.698 | 0.485 | |
| UTS2 | rs515830 | 7,923,586 | A | 0.029 | 0.038 | | -0.045 | | 0.104 | | 0.771 | 0.441 | |
| UTS2 | rs504560 | 7,926,542 | A | -0.059 | 0.034 | | -0.126 | | 0.008 | | -1.740 | 0.082 | |
| UTS2 | rs500508 | 7,927,456 | T | -0.059 | 0.034 | | -0.126 | | 0.008 | | -1.726 | 0.085 | |
| UTS2 | rs579992 | 7,927,981 | C | -0.034 | 0.056 | | -0.143 | | 0.076 | | -0.600 | 0.549 | |
| UTS2 | rs2066980 | 7,928,181 | G | 0.010 | 0.034 | | -0.056 | | 0.076 | | 0.301 | 0.764 | |
| UTS2 | rs2066978 | 7,928,759 | C | 0.059 | 0.037 | | -0.012 | | 0.131 | | 1.624 | 0.105 | |
| UTS2 | rs228725 | 7,929,819 | T | 0.009 | 0.032 | | -0.053 | | 0.071 | | 0.277 | 0.782 | |
| UTS2 | rs228724 | 7,930,554 | C | 0.009 | 0.032 | | -0.053 | | 0.071 | | 0.277 | 0.782 | |
| UTS2 | rs228721 | 7,931,588 | A | 0.027 | 0.037 | | -0.046 | | 0.099 | | 0.726 | 0.468 | |
| UTS2 | rs228720 | 7,933,457 | G | 0.009 | 0.032 | | -0.053 | | 0.071 | | 0.277 | 0.782 | |
| UTS2 | rs228719 | 7,934,171 | A | 0.009 | 0.032 | | -0.053 | | 0.071 | | 0.277 | 0.782 | |
| UTS2 | rs228716 | 7,936,272 | G | 0.009 | 0.032 | | -0.053 | | 0.071 | | 0.277 | 0.782 | |
| UTS2 | rs228714 | 7,938,648 | G | 0.009 | 0.032 | | -0.053 | | 0.071 | | 0.277 | 0.782 | |
| UTS2 | rs228703 | 7,944,264 | G | 0.014 | 0.030 | | -0.045 | | 0.073 | | 0.470 | 0.638 | |
| UTS2 | rs1040396 | 7,952,404 | C | -0.061 | 0.032 | | -0.125 | | 0.002 | | -1.897 | 0.058 | |
| UTS2 | rs1040397 | 7,952,427 | A | -0.061 | 0.032 | | -0.125 | | 0.002 | | -1.897 | 0.058 | |
| UTS2 | rs665244 | 7,970,248 | A | 0.038 | 0.056 | | -0.072 | | 0.148 | | 0.684 | 0.494 | |
| TNFRSF9 | rs2453021 | 7,989,566 | T | 0.031 | 0.032 | | -0.032 | | 0.094 | | 0.957 | 0.339 | |
| TNFRSF9 | rs863171 | 7,992,615 | T | 0.008 | 0.032 | | -0.055 | | 0.070 | | 0.239 | 0.811 | |
|  | rs2493215 | 8,007,716 | G | 0.002 | 0.031 | | -0.058 | | 0.063 | | 0.078 | 0.938 | |
|  | rs226474 | 8,009,763 | T | 0.002 | 0.031 | | -0.058 | | 0.063 | | 0.078 | 0.938 | |
| PARK7 | rs226249 | 8,021,778 | C | 0.032 | 0.032 | | -0.032 | | 0.095 | | 0.976 | 0.330 | |
| PARK7 | rs3766606 | 8,022,197 | T | -0.024 | 0.041 | | -0.104 | | 0.057 | | -0.574 | 0.566 | |
| PARK7 | rs226251 | 8,024,690 | T | 0.032 | 0.032 | | -0.032 | | 0.095 | | 0.976 | 0.330 | |
| PARK7 | rs7517357 | 8,025,275 | T | -0.024 | 0.041 | | -0.104 | | 0.057 | | -0.574 | 0.566 | |
| PARK7 | rs161802 | 8,042,826 | T | -0.014 | 0.041 | | -0.094 | | 0.066 | | -0.348 | 0.728 | |
| PARK7 | rs225119 | 8,044,361 | T | 0.020 | 0.031 | | -0.040 | | 0.081 | | 0.657 | 0.511 | |
|  | rs12727642 | 8,046,672 | A | -0.014 | 0.041 | | -0.094 | | 0.066 | | -0.348 | 0.728 | |
|  | rs17367289 | 8,053,135 | G | -0.014 | 0.041 | | -0.094 | | 0.066 | | -0.348 | 0.728 | |
|  | rs225100 | 8,066,914 | T | 0.020 | 0.031 | | -0.040 | | 0.081 | | 0.659 | 0.510 | |
| ERRFI1 | rs397349 | 8,074,872 | C | -0.009 | 0.040 | | -0.087 | | 0.069 | | -0.222 | 0.825 | |
| ERRFI1 | rs400736 | 8,078,309 | T | 0.020 | 0.031 | | -0.040 | | 0.081 | | 0.659 | 0.510 | |
| ERRFI1 | rs10489450 | 8,079,301 | T | -0.009 | 0.040 | | -0.087 | | 0.069 | | -0.222 | 0.825 | |
| ERRFI1 | rs442862 | 8,079,494 | T | 0.020 | 0.031 | | -0.040 | | 0.081 | | 0.659 | 0.510 | |
| ERRFI1 | rs28624 | 8,084,355 | C | -0.009 | 0.040 | | -0.087 | | 0.069 | | -0.222 | 0.825 | |
| ERRFI1 | rs408320 | 8,085,328 | T | 0.020 | 0.031 | | -0.040 | | 0.081 | | 0.659 | 0.510 | |
|  | rs225132 | 8,095,500 | G | -0.009 | 0.040 | | -0.087 | | 0.069 | | -0.222 | 0.825 | |
|  | rs6577459 | 8,100,173 | T | -0.039 | 0.045 | | -0.128 | | 0.049 | | -0.870 | 0.385 | |
|  | rs1883679 | 8,100,451 | G | -0.009 | 0.040 | | -0.087 | | 0.069 | | -0.222 | 0.825 | |
|  | rs2050198 | 8,111,839 | G | -0.009 | 0.040 | | -0.087 | | 0.069 | | -0.222 | 0.825 | |
|  | rs12753070 | 8,114,319 | G | 0.000 | 0.042 | | -0.083 | | 0.083 | | -0.005 | 0.996 | |
|  | rs4908724 | 8,119,251 | T | -0.033 | 0.044 | | -0.120 | | 0.054 | | -0.743 | 0.458 | |
|  | rs12748993 | 8,129,507 | G | -0.009 | 0.040 | | -0.087 | | 0.069 | | -0.222 | 0.825 | |
|  | rs12730860 | 8,132,462 | C | -0.009 | 0.040 | | -0.087 | | 0.069 | | -0.222 | 0.825 | |
|  | rs7539255 | 8,133,352 | C | -0.009 | 0.040 | | -0.087 | | 0.069 | | -0.222 | 0.825 | |
|  | rs12736494 | 8,136,016 | A | -0.009 | 0.040 | | -0.087 | | 0.069 | | -0.222 | 0.825 | |
|  | rs12758337 | 8,145,294 | T | -0.010 | 0.034 | | -0.076 | | 0.056 | | -0.302 | 0.763 | |
|  | rs11121086 | 8,151,224 | A | -0.010 | 0.034 | | -0.076 | | 0.056 | | -0.302 | 0.763 | |
|  | rs7553544 | 8,165,719 | A | -0.008 | 0.033 | | -0.073 | | 0.057 | | -0.236 | 0.814 | |
|  | rs10864330 | 8,168,564 | T | -0.008 | 0.033 | | -0.073 | | 0.057 | | -0.236 | 0.814 | |
|  | rs11121090 | 8,168,634 | T | -0.008 | 0.033 | | -0.073 | | 0.057 | | -0.236 | 0.814 | |
