# Supplementary material for: Association Analysis of Urotensin II Gene (UTS2) and Flanking Regions with Biochemical Parameters Related to Insulin Resistance
Source: PLoS One. 2011 Apr 29;6(4):e19327. doi: 10.1371/journal.pone.0019327 (PMC3084835; doi:10.1371/journal.pone.0019327)
Supplement: Table S4 — HOMA: genetic association analysis at UTS2 gene region. (DOC) [file pone.0019327.s004.doc]

Table S4. HOMA: genetic association analysis at *UTS2* gene region.

| **GENE** | **SNP** | **Bp (hg19)** | **A1** | | **BETA** | **SE** | **L95** | **U95** | **STAT** | **P** |
| --- | --- | --- | --- | --- | --- | --- | --- | --- | --- | --- |
| CAMTA1 | rs4908665 | 7,715,776 | | T | 0.000 | 0.037 | -0.073 | 0.072 | -0.006 | 0.995 |
| CAMTA1 | rs9434881 | 7,716,768 | | C | 0.000 | 0.037 | -0.073 | 0.072 | -0.006 | 0.995 |
| CAMTA1 | rs17031253 | 7,717,080 | | A | -0.024 | 0.040 | -0.102 | 0.055 | -0.591 | 0.555 |
| CAMTA1 | rs9434882 | 7,717,962 | | C | 0.000 | 0.037 | -0.073 | 0.072 | -0.006 | 0.995 |
| CAMTA1 | rs17376559 | 7,718,093 | | A | -0.024 | 0.040 | -0.102 | 0.055 | -0.591 | 0.555 |
| CAMTA1 | rs17031274 | 7,719,653 | | G | -0.024 | 0.040 | -0.102 | 0.055 | -0.591 | 0.555 |
| CAMTA1 | rs12071290 | 7,719,695 | | C | -0.022 | 0.040 | -0.101 | 0.056 | -0.550 | 0.582 |
| CAMTA1 | rs2995026 | 7,777,415 | | T | 0.053 | 0.057 | -0.058 | 0.164 | 0.932 | 0.352 |
| CAMTA1 | rs6693805 | 7,784,438 | | A | -0.083 | 0.047 | -0.176 | 0.010 | -1.755 | 0.080 |
| CAMTA1 | rs4908688 | 7,795,554 | | T | -0.083 | 0.047 | -0.176 | 0.010 | -1.755 | 0.080 |
| CAMTA1 | rs6577456 | 7,818,679 | | G | -0.084 | 0.035 | -0.153 | -0.015 | -2.372 | **0.018** |
| CAMTA1 | rs697672 | 7,826,347 | | C | 0.012 | 0.048 | -0.083 | 0.106 | 0.240 | 0.810 |
| CAMTA1 | rs41454244 | 7,829,286 | | C | 0.026 | 0.052 | -0.077 | 0.128 | 0.486 | 0.627 |
| VAMP3 | rs697674 | 7,837,878 | | G | -0.083 | 0.047 | -0.176 | 0.010 | -1.755 | 0.080 |
| VAMP3 | rs697675 | 7,838,113 | | C | -0.083 | 0.047 | -0.176 | 0.010 | -1.755 | 0.080 |
| PER3 | rs836755 | 7,846,527 | | C | -0.091 | 0.036 | -0.162 | -0.020 | -2.498 | **0.013** |
| PER3 | rs228727 | 7,847,836 | | C | -0.084 | 0.035 | -0.153 | -0.015 | -2.372 | **0.018** |
| PER3 | rs707463 | 7,850,062 | | T | -0.091 | 0.036 | -0.162 | -0.020 | -2.498 | **0.013** |
| PER3 | rs697686 | 7,850,218 | | T | -0.091 | 0.036 | -0.162 | -0.020 | -2.498 | **0.013** |
| PER3 | rs4908694 | 7,850,898 | | T | -0.083 | 0.047 | -0.176 | 0.010 | -1.755 | 0.080 |
| PER3 | rs696306 | 7,854,998 | | T | -0.088 | 0.037 | -0.160 | -0.016 | -2.382 | **0.017** |
| PER3 | rs1012477 | 7,858,135 | | C | -0.083 | 0.047 | -0.176 | 0.010 | -1.755 | 0.080 |
| PER3 | rs707465 | 7,861,304 | | C | -0.088 | 0.037 | -0.160 | -0.016 | -2.382 | **0.017** |
| PER3 | rs228641 | 7,862,899 | | T | 0.031 | 0.099 | -0.163 | 0.224 | 0.309 | 0.757 |
| PER3 | rs10864316 | 7,872,076 | | G | 0.065 | 0.047 | -0.027 | 0.158 | 1.384 | 0.167 |
| PER3 | rs4908482 | 7,877,488 | | A | -0.084 | 0.035 | -0.153 | -0.015 | -2.372 | **0.018** |
| PER3 | rs10746473 | 7,878,056 | | A | -0.084 | 0.035 | -0.153 | -0.015 | -2.372 | **0.018** |
| PER3 | rs12141033 | 7,878,547 | | A | -0.084 | 0.035 | -0.153 | -0.015 | -2.372 | **0.018** |
| PER3 | rs228688 | 7,879,130 | | T | -0.084 | 0.035 | -0.153 | -0.015 | -2.372 | **0.018** |
| PER3 | rs10462018 | 7,879,627 | | T | -0.083 | 0.047 | -0.176 | 0.010 | -1.755 | 0.080 |
| PER3 | rs228691 | 7,880,469 | | A | -0.084 | 0.035 | -0.153 | -0.015 | -2.372 | **0.018** |
| PER3 | rs10462020 | 7,880,683 | | G | 0.065 | 0.047 | -0.027 | 0.158 | 1.384 | 0.167 |
| PER3 | rs17374292 | 7,881,234 | | T | -0.083 | 0.047 | -0.176 | 0.010 | -1.755 | 0.080 |
| PER3 | rs228694 | 7,883,834 | | A | -0.084 | 0.035 | -0.153 | -0.015 | -2.372 | **0.018** |
| PER3 | rs697690 | 7,884,580 | | C | -0.089 | 0.037 | -0.161 | -0.017 | -2.432 | **0.015** |
| PER3 | rs17374439 | 7,888,438 | | T | 0.061 | 0.046 | -0.028 | 0.151 | 1.343 | 0.180 |
| PER3 | rs12061787 | 7,888,730 | | C | -0.102 | 0.049 | -0.198 | -0.006 | -2.073 | **0.039** |
| PER3 | rs228664 | 7,891,083 | | A | 0.080 | 0.106 | -0.129 | 0.288 | 0.750 | 0.454 |
| PER3 | rs12130462 | 7,891,378 | | T | 0.065 | 0.047 | -0.027 | 0.158 | 1.384 | 0.167 |
| PER3 | rs10462021 | 7,897,133 | | G | 0.065 | 0.047 | -0.027 | 0.158 | 1.384 | 0.167 |
| PER3 | rs12741937 | 7,897,622 | | T | -0.092 | 0.048 | -0.186 | 0.003 | -1.900 | 0.058 |
| UTS2 | rs228652 | 7,908,888 | | A | 0.057 | 0.038 | -0.017 | 0.130 | 1.507 | 0.132 |
| UTS2 | rs4908486 | 7,914,835 | | T | -0.013 | 0.036 | -0.084 | 0.058 | -0.352 | 0.725 |
| UTS2 | rs228637 | 7,917,632 | | A | 0.046 | 0.046 | -0.044 | 0.137 | 1.009 | 0.313 |
| UTS2 | rs17374781 | 7,919,363 | | C | -0.072 | 0.045 | -0.161 | 0.016 | -1.605 | 0.109 |
| UTS2 | rs531485 | 7,921,952 | | G | 0.038 | 0.041 | -0.043 | 0.118 | 0.909 | 0.363 |
| UTS2 | rs515830 | 7,923,586 | | A | 0.038 | 0.042 | -0.044 | 0.121 | 0.912 | 0.362 |
| UTS2 | rs504560 | 7,926,542 | | A | -0.079 | 0.038 | -0.153 | -0.004 | -2.078 | **0.038** |
| UTS2 | rs500508 | 7,927,456 | | T | -0.079 | 0.038 | -0.153 | -0.004 | -2.070 | **0.039** |
| UTS2 | rs579992 | 7,927,981 | | C | -0.030 | 0.062 | -0.152 | 0.092 | -0.480 | 0.632 |
| UTS2 | rs2066980 | 7,928,181 | | G | 0.020 | 0.038 | -0.053 | 0.094 | 0.535 | 0.593 |
| UTS2 | rs2066978 | 7,928,759 | | C | 0.080 | 0.041 | 0.000 | 0.159 | 1.964 | 0.050 |
| UTS2 | rs228725 | 7,929,819 | | T | 0.026 | 0.035 | -0.043 | 0.094 | 0.739 | 0.461 |
| UTS2 | rs228724 | 7,930,554 | | C | 0.026 | 0.035 | -0.043 | 0.094 | 0.739 | 0.461 |
| UTS2 | rs228721 | 7,931,588 | | A | 0.037 | 0.041 | -0.043 | 0.118 | 0.904 | 0.366 |
| UTS2 | rs228720 | 7,933,457 | | G | 0.026 | 0.035 | -0.043 | 0.094 | 0.739 | 0.461 |
| UTS2 | rs228719 | 7,934,171 | | A | 0.026 | 0.035 | -0.043 | 0.094 | 0.739 | 0.461 |
| UTS2 | rs228716 | 7,936,272 | | G | 0.026 | 0.035 | -0.043 | 0.094 | 0.739 | 0.461 |
| UTS2 | rs228714 | 7,938,648 | | G | 0.026 | 0.035 | -0.043 | 0.094 | 0.739 | 0.461 |
| UTS2 | rs228703 | 7,944,264 | | G | 0.013 | 0.033 | -0.052 | 0.079 | 0.402 | 0.688 |
| UTS2 | rs1040396 | 7,952,404 | | C | -0.067 | 0.036 | -0.137 | 0.004 | -1.859 | 0.063 |
| UTS2 | rs1040397 | 7,952,427 | | A | -0.067 | 0.036 | -0.137 | 0.004 | -1.859 | 0.063 |
| UTS2 | rs665244 | 7,970,248 | | A | 0.083 | 0.063 | -0.039 | 0.206 | 1.329 | 0.184 |
| TNFRSF9 | rs2453021 | 7,989,566 | | T | 0.040 | 0.036 | -0.030 | 0.111 | 1.121 | 0.263 |
| TNFRSF9 | rs863171 | 7,992,615 | | T | 0.020 | 0.035 | -0.049 | 0.089 | 0.565 | 0.573 |
|  | rs2493215 | 8,007,716 | | G | 0.018 | 0.034 | -0.049 | 0.086 | 0.533 | 0.594 |
|  | rs226474 | 8,009,763 | | T | 0.018 | 0.034 | -0.049 | 0.086 | 0.533 | 0.594 |
| PARK7 | rs226249 | 8,021,778 | | C | 0.042 | 0.036 | -0.028 | 0.112 | 1.170 | 0.242 |
| PARK7 | rs3766606 | 8,022,197 | | T | -0.032 | 0.046 | -0.122 | 0.057 | -0.712 | 0.477 |
| PARK7 | rs226251 | 8,024,690 | | T | 0.042 | 0.036 | -0.028 | 0.112 | 1.170 | 0.242 |
| PARK7 | rs7517357 | 8,025,275 | | T | -0.032 | 0.046 | -0.122 | 0.057 | -0.712 | 0.477 |
| PARK7 | rs161802 | 8,042,826 | | T | -0.023 | 0.045 | -0.112 | 0.065 | -0.515 | 0.607 |
| PARK7 | rs225119 | 8,044,361 | | T | 0.031 | 0.034 | -0.036 | 0.098 | 0.896 | 0.371 |
|  | rs12727642 | 8,046,672 | | A | -0.023 | 0.045 | -0.112 | 0.065 | -0.515 | 0.607 |
|  | rs17367289 | 8,053,135 | | G | -0.023 | 0.045 | -0.112 | 0.065 | -0.515 | 0.607 |
|  | rs225100 | 8,066,914 | | T | 0.031 | 0.034 | -0.036 | 0.097 | 0.897 | 0.370 |
| ERRFI1 | rs397349 | 8,074,872 | | C | -0.022 | 0.044 | -0.109 | 0.065 | -0.500 | 0.617 |
| ERRFI1 | rs400736 | 8,078,309 | | T | 0.031 | 0.034 | -0.036 | 0.097 | 0.897 | 0.370 |
| ERRFI1 | rs10489450 | 8,079,301 | | T | -0.022 | 0.044 | -0.109 | 0.065 | -0.500 | 0.617 |
| ERRFI1 | rs442862 | 8,079,494 | | T | 0.031 | 0.034 | -0.036 | 0.097 | 0.897 | 0.370 |
| ERRFI1 | rs28624 | 8,084,355 | | C | -0.022 | 0.044 | -0.109 | 0.065 | -0.500 | 0.617 |
| ERRFI1 | rs408320 | 8,085,328 | | T | 0.031 | 0.034 | -0.036 | 0.097 | 0.897 | 0.370 |
|  | rs225132 | 8,095,500 | | G | -0.022 | 0.044 | -0.109 | 0.065 | -0.500 | 0.617 |
|  | rs6577459 | 8,100,173 | | T | -0.057 | 0.050 | -0.156 | 0.041 | -1.143 | 0.254 |
|  | rs1883679 | 8,100,451 | | G | -0.022 | 0.044 | -0.109 | 0.065 | -0.500 | 0.617 |
|  | rs2050198 | 8,111,839 | | G | -0.022 | 0.044 | -0.109 | 0.065 | -0.500 | 0.617 |
|  | rs12753070 | 8,114,319 | | G | -0.017 | 0.047 | -0.109 | 0.075 | -0.357 | 0.721 |
|  | rs4908724 | 8,119,251 | | T | -0.049 | 0.049 | -0.146 | 0.047 | -1.001 | 0.317 |
|  | rs12748993 | 8,129,507 | | G | -0.022 | 0.044 | -0.109 | 0.065 | -0.500 | 0.617 |
|  | rs12730860 | 8,132,462 | | C | -0.022 | 0.044 | -0.109 | 0.065 | -0.500 | 0.617 |
|  | rs7539255 | 8,133,352 | | C | -0.022 | 0.044 | -0.109 | 0.065 | -0.500 | 0.617 |
|  | rs12736494 | 8,136,016 | | A | -0.022 | 0.044 | -0.109 | 0.065 | -0.500 | 0.617 |
|  | rs12758337 | 8,145,294 | | T | 0.009 | 0.037 | -0.064 | 0.082 | 0.237 | 0.813 |
|  | rs11121086 | 8,151,224 | | A | 0.009 | 0.037 | -0.064 | 0.082 | 0.237 | 0.813 |
|  | rs7553544 | 8,165,719 | | A | 0.010 | 0.037 | -0.062 | 0.083 | 0.275 | 0.783 |
|  | rs10864330 | 8,168,564 | | T | 0.010 | 0.037 | -0.062 | 0.083 | 0.275 | 0.783 |
|  | rs11121090 | 8,168,634 | | T | 0.010 | 0.037 | -0.062 | 0.083 | 0.275 | 0.783 |
