# Supplementary material for: Association Analysis of Urotensin II Gene (UTS2) and Flanking Regions with Biochemical Parameters Related to Insulin Resistance
Source: PLoS One. 2011 Apr 29;6(4):e19327. doi: 10.1371/journal.pone.0019327 (PMC3084835; doi:10.1371/journal.pone.0019327)
Supplement: Table S5 — Genotyped polymorphisms included in the Affymetrix 250 k NspI chip. (DOC) [file pone.0019327.s005.doc]

Table S5. Genotyped polymorphisms included in the Affymetrix 250k NspI chip.

| **Probe Set ID** | **dbSNP RS ID** |
| --- | --- |
| SNP_A-1785925 | rs228694 |
| SNP_A-1786989 | rs697672 |
| SNP_A-1792597 | rs17374439 |
| SNP_A-1795952 | rs2453021 |
| SNP_A-1811003 | rs531485 |
| SNP_A-1837515 | rs28624 |
| SNP_A-1837974 | rs1040396 |
| SNP_A-1904682 | rs579992 |
| SNP_A-1942079 | rs500508 |
| SNP_A-2021270 | rs4908486 |
| SNP_A-2040833 | rs697690 |
| SNP_A-2068873 | rs228688 |
| SNP_A-2071607 | rs697686 |
| SNP_A-2135045 | rs41454244 |
| SNP_A-2140825 | rs17376559 |
| SNP_A-2153792 | rs2493215 |
| SNP_A-2160093 | rs707463 |
| SNP_A-2181217 | rs228652 |
| SNP_A-2218777 | rs2066978 |
| SNP_A-2236124 | rs9434882 |
| SNP_A-2251186 | rs225100 |
| SNP_A-2256380 | rs228703 |
| SNP_A-2302021 | rs10462018 |
| SNP_A-4213897 | rs836755 |
| SNP_A-4224703 | rs397349 |
| SNP_A-4229050 | rs228721 |
